# Human resources for health in Peru: recent trends (2007–2013) in the labour market for physicians, nurses and midwives

**DOI:** 10.1186/s12960-017-0243-y

**Published:** 2017-09-21

**Authors:** M. Michelle Jimenez, Anthony L. Bui, Eduardo Mantilla, J. Jaime Miranda

**Affiliations:** 1 CRONICAS Centre of Excellence in Chronic Diseases, Cayetano Heredia University, Av. Armendáriz 497, Miraflores, Lima 18, Peru; 2 David Geffen School of Medicine at UCLA, Los Angeles, United States of America; 3 ESAN University, Lima, Peru

**Keywords:** Human resources for health, Labour market, Peru

## Abstract

**Background:**

Most analyses of gaps in human resources for health (HRH) do not consider training and the transition of graduates into the labour market. This study aims to explore the labour market for Peru’s recent medical, nursing, and midwifery graduates as well as their transition into employment in the Ministry of Health’s (MOH) system.

**Methods:**

Data from four different datasets, covering 2007–2013, was used to characterize the patterns of recently trained physicians, nurses, midwives, and postgraduate-trained physicians that enter employment in the MOH system, and scenario analyses were used to describe how this rate of entry needs to adapt in order to fill current HRH shortages.

**Results:**

HRH graduates have been increasing from 2007 to 2011, but the proportions that enter employment in the MOH system 2 years later range from 8 to 45% and less than 10% of newly trained medical specialists. Scenario analyses indicate that the gap for physicians and nurses will be met in 2027 and 2024, respectively, while midwives in 2017. However, if the number of HRH graduates entering the MOH system doubles, these gaps could be filled as early as 2020 for physicians and 2019 for nurses. In this latter scenario, the MOH system would still only utilize 56% of newly qualified physicians, 74% of nurses, and 66% of midwives available in the labour market.

**Conclusion:**

At 2013 training rates, Peru has the number of physicians, nurses, and midwives it needs to address HRH shortages and meet estimated HRH gaps in the national MOH system during the next decade. However, a significant number of newly qualified health professionals do not work for the MOH system within 2 years of graduation. These analyses highlight the importance of building adequate incentive structures to improve the entry and retention of HRH into the public sector.

## Background

Changes in demography, epidemiology, and disability burden patterns create increasing pressure on healthcare systems and the roles of healthcare professionals [1]. In order to achieve universal health coverage, countries need to train, incorporate, and retain enough healthcare professionals into their health systems to meet the needs of the whole population. Peru, a country facing a double burden of communicable and non-communicable diseases [2], still faces gaps in access to healthcare related in part to difficulties in the recruitment and retention of health workers in rural and remote regions [3].

The World Health Organization has defined the minimum threshold to provide essential maternal and child healthcare services as 23 physicians, nurses, and midwives per 10 000 population [4], as a lower density of healthcare professionals has been associated with increased maternal, infant, and under-five mortality rates [5]. While Peru met the overall threshold for human resources for health (HRH) density at 29.6 in 2015, there remains a distribution problem, with a high concentration of HRH in larger metropolitan areas such as Lima, Callao, and Arequipa [6]. However, the classical analysis of shortages of HRH based on their density per population has its shortcomings in that it does not consider the labour market perspective [7], such as the supply end (training of graduates), nor the transition of labour into and outside of the system, including migration. Peru has seen a rise in medical schools, 33 in 2010 compared to 3 in 1960, and changes to healthcare workers contracts in 2008—with a shift to more temporary ones, making it harder to secure permanent positions [8]. Exploring the labour market for Peru’s recent medical, nursing, and midwifery graduates can provide better understanding of the patterns of Peru’s HRH shortage.

Benefiting from access to primary data sources, this study has three objectives: (1) to quantify how many recently trained physicians, nurses, and midwives enter the national health system of the Ministry of Health (MOH); (2) to describe how the rate of entry into Peru’s health sector should adapt in order to fill the HRH gap in physicians, nurses, and midwives at the primary care level, [NB: this gap has been defined by the MOH [9] as a difference between needs and availability, the former calculated by estimating population catchment areas and demand for medical services listed in the Essential Health Insurance Plan, and the latter as provided by the National Observatory for HRH]; and (3) to quantify how many postgraduate-trained physicians in specialties deemed as priority by the MOH (anaesthesiology, family and community medicine, general surgery, gynaecology and obstetrics, internal medicine, and paediatrics) enter the MOH’s public health system.

### Context

**Fig. 1:**
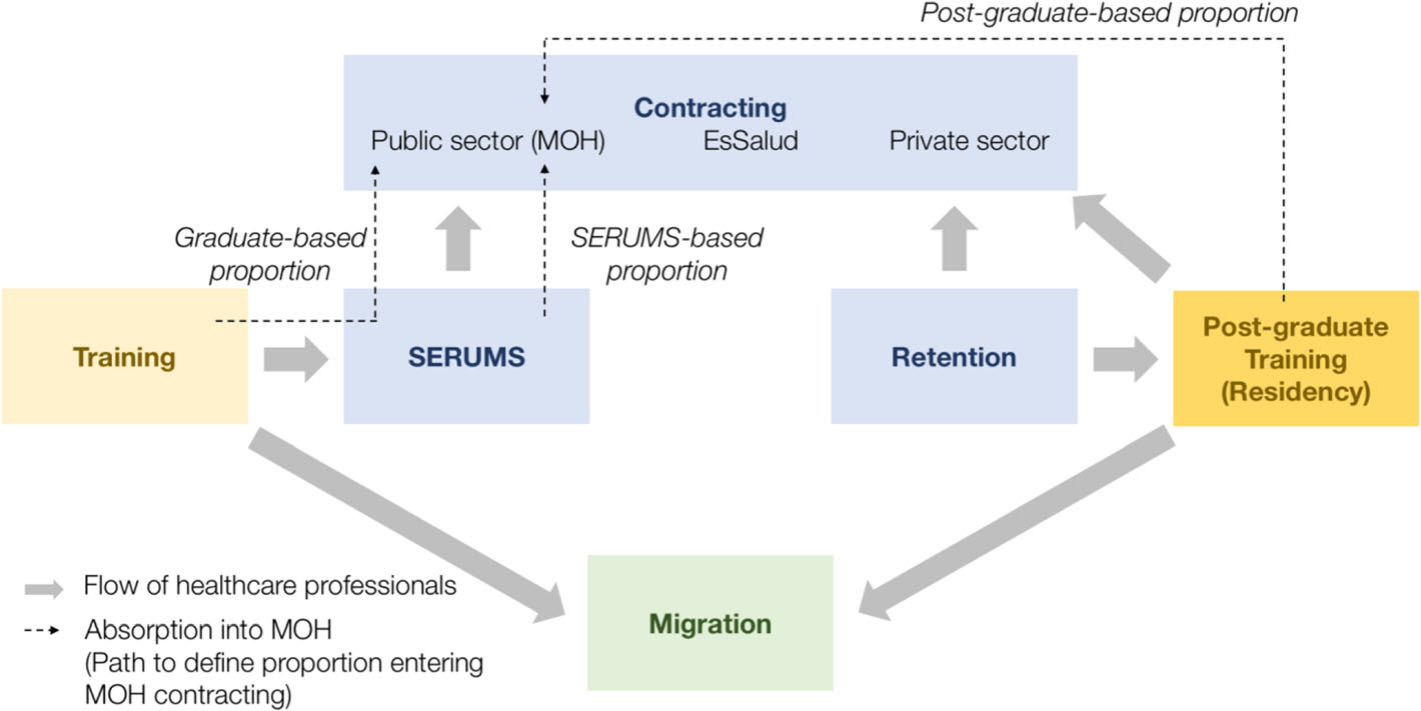
Conceptual framework of the study

Healthcare in Peru is provided by the MOH, ESSALUD, Armed Forces and National Police, and the private sector. The publicly funded MOH system cares for approximately 60% of the population, while ESSALUD, a contribution-based social security system, covers approximately 30% [8, 10]. Figure 1 presents this study’s conceptual framework showing the labour market for HRH in Peru, focusing on the MOH’s system only. After training, HRH interested in pursuing employment or postgraduate training within the MOH’s system must first spend 1 year completing the Urban Rural Marginal Internship Program (SERUMS), working at the primary care level in rural or urban-marginal areas. After completing SERUMS, professionals may continue to work for the MOH system or join other healthcare sectors and may apply to various universities offering training positions within hospitals for postgraduate specialist training.

In terms of the offer of training for health professionals, there was a significant increase in the number of private universities created during the 1990s in Peru. Thus, the offer of university-level training has increased significantly [8]. At the same time, the number of State-funded universities and their ability to train professionals has not expanded significantly in the last decades.

## Methods

### Data sources

This study used four databases: (a) graduate numbers from university training programmes for physicians, nurses, and midwives for 2007–2011 were obtained from the National Assembly of Deaneries (detailed analyses on these data have been published previously [11]); (b) applicants, entrants, and graduates of SERUMS for 2007–2013; (c) MOH’s National Observatory for HRH including data on individual employment status and titles for 2007, 2009, 2010, and 2012, both provided by the Ministry of Health’s HRH Management and Development Directorate; and (d) entrants into postgraduate clinical specialties deemed as priority by the MOH for 2009–2010 provided by the National Committee for Medical Residencies. This study excludes ESSALUD, Armed Forces and National Police, and the private sector due to lack of data on number and type of HRH working in these sectors.

### Analysis

#### Graduates entering the national health system

The numbers of university graduates from 2007 to 2011 was compared to the numbers of SERUMS healthcare workers in the immediate subsequent year, i.e. 2008 number of SERUMS was compared to the number of 2007 graduates, reflecting five cohorts of graduates, to calculate the proportion of graduates that begin SERUMS immediately after completing university. The transition of SERUMS graduates to employment status within the MOH system was analysed by matching individuals’ names in the SERUMS database covering 2007–2012 with the National Observatory of HRH database as of September 2013. The matching was done by staff at the MOH’s National Observatory of HRH, and the study team received an anonymised dataset. For each of the five annual cohorts, a SERUMS-based proportion was calculated as the fraction of SERUMS healthcare workers that enter the MOH system in the following year, as well as an overall graduate-based proportion of those entering the MOH system, dividing the number of new MOH healthcare workers by the total number of graduates 2 years prior.

#### Scenarios for closing the HRH gaps

Gaps in HRH and supply of new MOH healthcare workers were projected from 2013 through 2050 by combining MOH-estimated gaps in HRH, projections of population growth derived from the National Institute of Statistics and Informatics, and the number of healthcare professionals that would graduate in the coming years, by applying the average ratio of graduates to students in the period 2007–2011 to the number of students entering university in 2008–2011 and assuming this ratio remained constant. Other assumptions were that starting in 2016, the number of professionals taking up SERUMS positions would stay constant and be equal to the projections for that year. Even though an increase in the number of new professionals could be expected, there are also a number that are lost every year; thus, it was assumed that the number of additional entries into SERUMS would balance the number of those who quit SERUMS. It was also assumed that the proportion of professionals who keep working in the MOH system remained constant. Analysis of scenarios was then varied by SERUMS-based proportions. Tables of projected gaps have been published elsewhere [12].

#### Postgraduate physicians entering the national health system

Physicians pursuing postgraduate clinical specialization do so as part of a national residency programme in hospitals across the Peruvian health sector, with the majority available at MOH and ESSALUD hospitals and thus financed mainly by the Government. Data on physicians entering postgraduate training in MOH hospitals during 2009–2010 was compared to the number of medical specialists entering the MOH system in 2012 and 2013 to assess whether the numbers of potential new specialists available in Peru are reflected in the number of specialists entering the public sector, once their training is finished. A postgraduate-based proportion was calculated by dividing the number of specialists hired by the MOH system in 2012 and 2013 to the number of entrants into postgraduate clinical training for MOH-priority specializations in 2009 and 2010.

## Results

### Flow of healthcare professional graduates

**Fig. 2:**
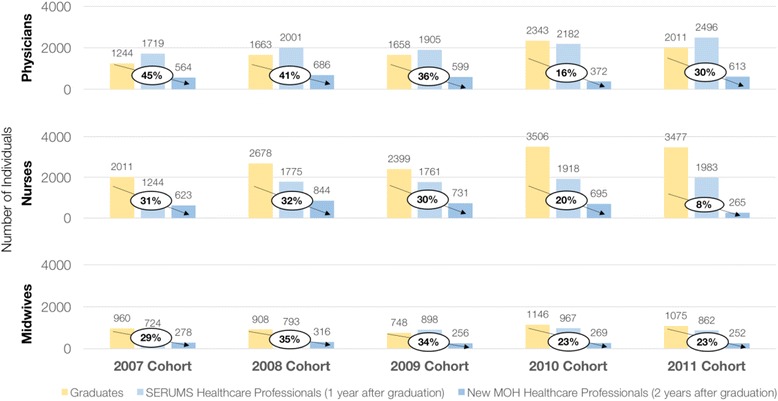
Flow of health professional graduates. Notes: Proportion in oval reflects graduate-based proportion, which is the number of graduates absorbed into the MOH system two years after graduating. Data used here is not cohort data, but is grouped visually and analytically to assume the fastest path to public sector, that is, working in SERUMS directly after graduating, and entering MINSA directly after SERUMS. The high uptake of physicians is a result of having more SERUMS health professionals than medical school graduates from the preceding year, which occurred due to increased demand for starting SERUMS and the backlog of graduates who were unable to obtain positions in previous years

Figure 2 shows the number of graduates from 2007 to 2011, the number of SERUM positions that were filled in the immediate year after (2008–2012), and those individuals who, after completing their SERUMS, were employed by the MOH system in the following year (2009–2013). Overall, both the number of graduates and the number of SERUMS healthcare professionals across physicians, nurses, and midwives has increased throughout this period, due in part to an MOH strategy to increase the availability of SERUMS positions to staff rural and remote health centres [9]. In turn, following graduation and SERUMS service, while the number of physicians entering the MOH system has slightly increased from 2007 to 2011, the number of nurses and midwives slightly decreased during the same time period.

In terms of the proportion of graduates who enter SERUMS, all physicians tend to do so, compared to an average of 62% for nurses and 85% for midwives over the time period. The arrows displayed on the figure depict the proportion of graduated professionals who become employed by the MOH system 2 years later, indicating the entry from the total pool of newly trained HRH available. However, it is the proportion of professionals that have completed their SERUMS and then become employed that shows the actual entry rate, given that a complete SERUMS is a prerequisite for employment in the MOH system. Using proportions of SERUMS-completed HRH that enter the MOH system (data not shown), the average uptake of employment over the time period was 28% for physicians, 37% for nurses, and 33% for midwives.

### Closing the HRH gap—a scenario analysis

Table 1 presents nine different scenarios in which the MOH system can fill its gaps of physicians, nurses, and midwives. Proportions of SERUMS professionals who enter the MOH as new hires are provided, as well as the year that the gap for a HRH role would be met assuming these proportions stayed constant. Under the current rate of entry into MOH employment, the gap in physicians would be met in 2027, nurses in 2024, and midwives in 2017. By doubling the SERUMS-based proportions, gaps would be filled as early as 2020 and 2019 for physicians and nurses, respectively. In this latter scenario, of the total of newly qualified HRH available in the Peruvian labour market, the MOH system would still utilize only 56% of physicians, 74% of nurses, and 66% of midwives. By decreasing its SERUMS-based proportion by 20% (scenario 8), the gaps would be filled several years later, 2030 for physicians, 2027 for nurses, and 2018 for midwives.

**Table 1:** Closing the gap

		Current situation 0%^b^	Scenario 1+ 10%^b^	Scenario 2+ 20%^b^	Scenario 3+ 30%^b^	Scenario 4+ 40%^b^	Scenario 5+ 50%^b^	Scenario 6+ 100%^b^	Scenario 7− 10%^b^	Scenario 8− 20%^b^
Physicians	Proportion^a^	0.28	0.31	0.34	0.36	0.39	0.42	0.56	0.25	0.22
	Year gap is met	2027	2025	2024	2024	2023	2022	2020	2028	2030
Nurses	Proportion^a^	0.37	0.41	0.44	0.48	0.52	0.56	0.74	0.33	0.30
	Year gap is met	2024	2023	2023	2022	2021	2021	2019	2026	2027
Midwives	Proportion^a^	0.33	0.36	0.40	0.44	0.46	0.50	0.66	0.30	0.26
	Year gap is met	2017	2017	2017	2017	2016	2016	2016	2018	2018

^a^Proportion listed is SERUMS-based proportion, i.e. percentage of SERUMS health professionals that enter as new MOH hires

^b^Percent change of SERUMS-based proportion from current situation’s proportion, which is the average SERUMS-based proportion from 2007 to 2011

### Entry of medical specialists into the MOH system

Table 2 shows the number of entrants into medical residency specialties in 2009 and 2010 and compares these to the number of specialists hired by the MOH system in 2012 and 2013, when these entrants would have completed their residency training. Specialists that completed their training by 2012 and 2013 would be eligible for MOH employment. Results show that only 9.9% of the total specialists were hired by the MOH system in those years. General surgeons, paediatricians, and anaesthesiologists joined the MOH system in higher numbers, but only one family and community medicine specialist joined the MOH system in 2013.

**Table 2:** Absorption of 2009–2010 medical residents in the public sector, 2012-2013

	Admissions to residency			Recent graduates of residency entering MOH			Postgraduate-based proportion of MOH entrants (%)
	2009	2010	Total	2012	2013	Total	
General surgery	65	78	143	20	8	28	19.6
Paediatrics	88	118	206	22	15	37	18.0
Anaesthesiology	72	87	159	23	5	28	17.6
Internal medicine	59	78	137	11	5	16	11.7
Gynaecology and obstetrics	87	117	204	16	4	20	9.8
Family and community medicine	33	42	75	0	1	1	1.3
Overall total	924	1156	2080	148	58	206	9.9

## Discussion

This study reports on a particular point in the cycle of HRH planning: the transition from training to employment in the national health system, in a country where this sector provides care for 60% of the population. Results from this analysis of primary data show that at 2013 training rates, Peru has the number of physicians, nurses, and midwives it needs to meet the estimated HRH gap in the national MOH system during the next decade. This is explained by the number of HRH graduates, which has been increasing from 2007 to 2011 due to the additional offer of training provided by private universities [11]. Yet, our analyses show that not only is availability of new graduates important but that current entry rates of HRH into employment by the MOH system may hinder meeting such targets. The proportion of HRH graduates entering the MOH system 2 years later ranges from 8 to 45% during the period of this study, showing that a significant number of qualified professionals do not enrol in the MOH system workforce in the immediate year after completing their SERUMS. Scenario analyses indicate that if 2013 absorption rates are held, the gap for physicians and nurses will not be met until 2027 and 2024, respectively, while the gap for midwives will likely be met by 2017. Furthermore, less than one in five newly trained MOH-priority medical specialists enrol in the MOH system workforce, suggesting that there are some major barriers to entry immediately after SERUMS and after completion of postgraduate clinical training.

Shortages in HRH have been reported in Peru indicating substantial deficiencies in the total number of available healthcare professionals and medical specialists [8, 13]. Using a labour market framework, the shortage of healthcare professionals in Peru may be explained by the low proportions of healthcare professionals entering the MOH system from SERUMS or from postgraduate clinical training shown in this study, high rates of out-migration [14], and low rates of retention rather than merely a limited supply in training of professionals [11].

The differences in the proportion of graduates who enter SERUMS (nearly 100% of physicians vs. 62% of nurses and 85% of midwives over the time period) in the immediate year after graduation could indicate that nurses and midwives may have other options of immediate employment in the private sector or the social security system that do not require being posted in a remote and rural area and may even offer longer-term opportunities than the MOH.

The average proportion of SERUMS healthcare professionals entering the MOH system has been consistently low, 28% of physicians, 38% of nurses, and 33% of midwives. To increase the number of healthcare professionals in the MOH system, strategies to raise the proportion of SERUMS-completed healthcare professionals and graduated medical specialists entering the MOH system need to be explored. In 2009, the Peruvian government instituted a strategy to increase the number of SERUMS positions at rural and remote outposts available to physicians [8], and while this indeed increased the number of SERUMS physicians, as seen in Fig. 2, these results show that it did not ultimately translate into more healthcare professionals remaining within the MOH system immediately after completing their SERUMS.

Entering employment in the MOH system from SERUMS is clearly not linear, but further investigation is needed to understand the specific barriers such as those on the supply side (e.g. lack of MOH system positions, mismatch between skills [11]) or the demand side (e.g. salary, work hours, prestige, professional development opportunities). Miranda et al. [15] and Huicho et al. [16] found that in Ayacucho, Peru, physicians were five times more likely, and nurses and midwives 14 times, to choose an urban-based job over a rural one. Incentives that professionals preferred included salary increases and bonus points to gain entry into postgraduate training programmes. For example, Mayta-Tristán et al. [17] proposed that SERUMS should be voluntary and not time-limited, offer academic incentives for further training after 3 years of service, and ensure basic workplace protection for the HRH. These proposals are modelled on the successful Rural Practitioner Program in Chile, a programme with a near 100% retention rate [18]. Nevertheless, recent reviews of recruitment and retention strategies for primary care physicians have found weak evidence [19, 20] except for some limited effectiveness of undergraduate and postgraduate placements in underserved areas and selective recruitment of medical students (i.e. those from rural areas) [19]. Similarly, the low proportions of medical specialists entering the MOH system after postgraduate clinical training not only further exacerbate the workforce shortage but indicate a significant loss of training investment. Although some of these losses can be explained by migration [14], further research is needed to evaluate the proportion of medical specialists who enter the MOH system in future years and explore possible barriers of entry, including availability of employment offers and their geographical distribution.

In addition to the low proportions of SERUMS-completed healthcare professionals and medical specialists entering the MOH system, the workforce shortage is compounded by mismatched training competency. Peru has been able to meet its demand for training in healthcare professions, with 70% of graduates attending private universities, yet a massive disconnect remains between the primary care-level competencies developed in universities and those desired by the MOH [11]. This mismatch continues for medical specialists in primary care with only one joining the MOH system in 2013 and low demand for postgraduate training positions within the MOH system (251 available in 2013 and only 143 taken up) [6].

These findings can be relevant to health systems in other countries, where quantity of HRH may not be critical but other constraints and barriers, fiscal or otherwise, exist [21]. For example, Peru has sufficient number of medical schools, 33 vs. 20 in Australia and 25 in England [9, 22, 23], and students to meet the physician gap. Yet, these results indicate that there are several points postgraduation (nurses and midwives) and post-SERUMS (all professionals) in which the system fails to attract and/or retain the professionals required to counteract HRH shortages. Countries that are investing in the development of their HRH should consider also investing in understanding health labour market trends [7], such as the rates of entry to employment at different points in a healthcare professional’s trajectory and determining the barriers to entering the public national health system. Building adequate incentive structures to improve the entry and retention of HRH into the public sector will require an evaluation of the value provided by already committed resources, including their cost-effectiveness [22] to ensure the best use of limited resources.

This study provides an assessment of the transition of healthcare professionals from university training, through SERUMS, and entry into the MOH system. It moves beyond merely quantifying HRH and signalling gaps in density to explore what happens to HRH within the local labour market and incorporates the complexity of various transition points. The main strength of this study included the ability to track individuals over time to assess if they ultimately become employed by the MOH system in the immediate year after completing their SERUMS, providing a robust exploration of trends over the time period. It makes use of primary data, rather than models or theoretical assumptions, and as such could be considered innovative. The focus of the study has international significance in relation to the market for physicians, nurses, and midwives in Peru, particularly in attracting new graduates into employment in the Ministry of Health’s (MOH) system. Nevertheless, this study has limitations. Data on HRH in this study accounts for those working for the MOH system, excluding those working for other sectors, such as ESSALUD and the private sector. Yet, because the MOH system covers the majority of the population, these findings still show evidence of matters causing the gap in healthcare professionals. Furthermore, the MOH’s databases shared with the authors did not include information on salaries, and no primary data sources on levels of HRH unemployment and migration existed to the knowledge of the authors. Projections were based on average rates for a period of 5 years. The analysis looked at the transition of HRH from graduation, to SERUMS, and then employment in the MOH system in the immediate years after but did not consider that professionals may join further on. However, it can be assumed that those employed by the MOH system are not employed by ESSALUD at the same time (as this is not allowed by the Government), although they might have a dual practice in the private sector. In spite of its limitations, the scenario projections included in this study may prove useful to policymakers in Peru and other countries in the same stage of development, to persuade them to use concurrent and synergic strategies to address gaps in HRH.

## Conclusion

Peru faces significant HRH shortages, and an analysis of its labour market shows a complex pattern with high availability of newly graduated HRH, and yet low entry into employment structures of the MOH system. These analyses provide evidence of the importance of understanding health labour market trends as a basis to build incentive structures for HRH to join the public national health system, not only in Peru but also in other countries investing in the development of their HRH.
